# Can Breastfeeding Prevent Long-Term Overweight and Obesity in Children? A Population-Based Cohort Study

**DOI:** 10.3390/nu16162728

**Published:** 2024-08-16

**Authors:** Carolina Roldão, Rita Lopes, Joana Matos Silva, Natália Neves, Joana Costa Gomes, Cristina Gavina, Tiago Taveira-Gomes

**Affiliations:** 1 Faculty of Medicine, University of Porto, 4200-319 Porto, Portugal; joanamatosdasilva@gmail.com (J.M.S.); natalineves@gmail.com (N.N.); cristina.gavina@gmail.com (C.G.); 2 MTG Research and Development Laboratory, 4200-604 Porto, Portugal; rita.lopes@mtg.pt (R.L.); tiagogomes@med.up.pt (T.T.-G.); 3 USF Caravela, Matosinhos Local Health Unit, 4460-352 Sra. da Hora, Portugal; joanacostabgomes@gmail.com; 4 The School of Medicine and Biomedical Sciences; University of Porto, 4050-313 Porto, Portugal; 5 Pedro Hispano Hospital—ULS Matosinhos, 4464-513 Matosinhos, Portugal; 6 The Cardiovascular R&D Unit (UnIC), Faculty of Medicine, University of Porto, 4200-319 Porto, Portugal; 7 Faculty of Health Sciences, Fernando Pessoa University, 4249-004 Porto, Portugal; 8 Department of Community Medicine, Health Information and Decision, Faculty of Medicine, University Porto, 4200-319 Porto, Portugal; 9 Center for Health Technology and Services Research (CINTESIS), 4200-450 Porto, Portugal

**Keywords:** breastfeeding, obesity, overweight, childhood

## Abstract

The aims of this study were to assess the impact of exclusive breastfeeding up to 6 months of age on reducing the incidence of overweight and obesity in children up to 10 years of age and to estimate the annual incidence of obesity and overweight in the study population. Our retrospective cohort analysis using electronic health records included children from zero to ten years old, born between 1 January 2006 and 31 December 2022, followed up at the Unidade Local de Saúde de Matosinhos (ULSM). Information on their comorbidity history was collected, and positive or negative control results were defined. In the first year of life, around 29% of the children on exclusive breastfeeding were obese and 20% were overweight. This trend was reversed by the age of 9. Asthma and allergic rhinitis were used as positive control outcomes and allergic dermatitis as a negative control outcome. There seems to be no relationship between exclusive and non-exclusive breastfeeding and the development of overweight or obesity at the age of 10. The results showed that breastfeeding is associated with a lower risk of asthma in the future.

## 1. Introduction

Breastfeeding (BF) is considered one of the cornerstones for promoting and protecting the health of children worldwide. The European Society for Pediatric Gastroenterology, Hepatology, and Nutrition has described BF as the most natural and recommended method for the growth and development of children [1].

The World Health Organization (WHO) recommends that children begin BF within the first hour of birth and be exclusively fed for the first 6 months of life alongside the introduction of nutritionally adequate and safe complementary (solid) food up to 2 years of age [2,3,4,5,6,7]. BF is the ideal means of healthy growth and development; it contains the nutrients needs for the development of the immature gastrointestinal system, central nervous system, endocrine system, and immune system [3].

The prevalence of BF has evolved over the years in Portugal. According to the EPACI 2012 study, 91% of Portuguese children started BF; however, this trend tended to decrease to 66% at 4 months of age, 53% at 6 months, 23% at 12 months, and just 3% at 24 months of age, with 33% of children exclusively breastfed up to 4 months of age and 21% up to 6 months of age. A greater probability of initiating BF was found in mothers with a higher level of education, higher monthly income, and who were not obese [8].

A single-centre study carried out in a Portuguese hospital maintained the same trend of results, with 80.5% of newborns starting exclusive BF, but a lower prevalence in those born by caesarean section (69.7%). During the second month of life, the prevalence of exclusive BF fell to 59.7% of the sample, with no significant differences between the different modes of delivery. The importance of family support after hospital discharge was emphasized [9].

It is believed that the type of nutrition in early life is critical for childhood obesity development [1].

The “Developmental Origins of Health and Disease” hypothesis argues that the environment at the beginning of life, namely the diet to which the child is exposed, can have an impact on the risk of chronic diseases in adult life [10]. For the effects of early exposure to persist over time, the exposure must leave some type of “mark” on the body [4]. It is believed that breast milk is capable of inducing epigenetic modifications, such as DNA methylation, mitigating the development of diseases such as overweight and obesity from infancy to early childhood [11]. Adverse exposure early in life results in epigenetic changes that may contribute to an increased risk of chronic diseases later in life such as type 2 diabetes and cardiovascular disease [10]. The greatest benefit of BF in preventing overweight and obesity in children is achieved if its consumption is continued for at least 6 months and extended until 2 years of age [2,3,5,6].

The prevention of childhood obesity is crucial in order to address this condition as a public health issue since we know that around 41 million children are overweight or obese [12].

Between the first (2008) and fifth (2019) rounds of the Childhood Obesity Surveillance Initiative (COSI) Portugal study, the trend in the prevalence of overweight and obesity in children reversed slightly. Then, between 2019 and 2022, this trend changed, with an increase of 2.2% in the prevalence of overweight (29.7% to 31.9%) and 1.6% in the prevalence of obesity (11.9% to 13.5%) in children [13].

In a study involving four European countries, including Portugal, it was found that the prevalence of overweight/obesity in children aged 4 to 13 who were breastfed between 3 and 6 months was lower than in children who were breastfed for more than 6 months [14]. As for body fat mass, 13-year-olds who were breastfed for less than 1 month had higher levels compared to children who were breastfed for more than 6 months. When breastfed from 3 to 6 months of age, they had lower levels of body fat mass compared to their peers who were breastfed for more than 6 months [14].

Thus, children’s eating behaviours seem to contribute to weight development. Children who were breastfed for the first 6 months of life were less likely to show a desire to drink sugary drinks and those who were breastfed for longer showed less food reactivity and desire to drink [15].

It is believed that a calm environment during breastfeeding makes mothers more sensitive to the infant’s hunger and satiety signals. More sensitive and less controlling behaviours during feeding allow infants to self-regulate their energy intake and learn to respond to internal hunger and satiety signals [16,17]. However, most of the literature has been based solely on the testimony of maternal feeding practices, and there are still few prospective studies explaining how breastfed children manage to control the desire to drink [16].

The relationship between BF and binge eating behaviour also might in theory be reduced by bioactive factors present in breast milk, such as appetite-regulating hormones: leptin, adiponectin and ghrelin. These hormones are involved in appetite regulation by preventing excess energy intake, in turn reducing the risk of obesity in the long term [15,17]. 

For example, ghrelin, by potentiating the secretion of growth hormone, showed an inverse relationship with weight gain among breastfed children, which was not observed among formula-fed children. However, more research is needed to clarify the underlying mechanisms that support the beneficial role of breastfeeding in the development of obesity [17]. 

Other studies have also concluded that breast milk can reduce early-life weight gain, the risk of overweight/obesity, and abdominal adiposity, with exclusive BF resulting in 20% slower weight gain rates compared to infants formula fed [8]. Evidence indicates that BF versus formula/mixed feeding or a longer BF duration is associated with lower Body Mass Index (BMI) trajectories, reducing the likelihood of overweight/obesity in children, proving the importance of promoting and continuing BF to support the prevention of childhood obesity [17,18].

However, the evidence still shows inconsistent results between the duration of BF in childhood and the reduction in excess weight/obesity in childhood/adolescence, requiring further studies [19,20,21,22].

The main goal of this study was to evaluate the impact of exclusive BF up to 6 months of age on reducing the incidence of overweight and obesity among children aged up to 10 years, in a northern Portuguese population. Furthermore, we estimated the annual incidence of obesity and overweight in the study population.

## 2. Materials and Methods

### 2.1. Study Design

A retrospective cohort analysis was carried out using electronic health records of children followed at the Unidade Local de Saúde de Matosinhos (ULSM) in child health consultations. ULSM is a large healthcare institution that includes 14 primary healthcare units supported by 1 hospital (Pedro Hispano Hospital), which provides services to the region of Matosinhos.

The present study included children born between 1 January 2006 and 31 December 2022, from zero to ten years of age, from the ULSM population, who did or did not receive exclusive BF until six months of age. The index date was defined as the first visit immediately after 6 months of age. The child health surveillance visit is intended for monitoring of children and young people under the age of 18 and must adhere to the surveillance schedule recommended by the National Health Plan. Children and adolescents are seen at key ages (0, 1, 2, 4, 6, 9, 12, 15, and 18 months and 2, 3, 4, 5, 6/7, 8, 10, 12–13, and 15–18 years old). From 2015, information from nursing and medical appointments has been recorded in the child health programme, with compliance with indicators defined in the BI-CSP [23].

The data are recorded by the nurse in the Child Health programme (SClinico^®^ software version 2.8.0) in specific fields for the height/length, weight, and BMI variables. At medical appointments, growth and development are evaluated, encouraging health-promoting behaviours. The type of BF is registered in the parameters to be evaluated at each appointment or registered in the doctor’s clinical record.

This study was approved by the Ethical Committee of ULSM (Nº 74/CES/JAS, 14 July 2023). The treatment and analysis of the data were conducted using analytical programs developed for these purposes and sent for execution at the ULSM data centre. Only aggregated data were shared with the investigators. Following the Health Insurance Portability and Accountability Act security standard, the data were de-identified by the ULSM Information Technology Department prior to running the analytic code.

### 2.2. Key Variable Definition

The study variables were weight (in kg), height (in m), length (in cm), and BMI (in kg/m^2^) for the study group (exclusive BF) and the control group (non-exclusive BF). The time points for determining the variables were set annually from 6 months of age to 10 years of age. Children born between 1 January 2006 and 31 December 2022 (inclusive) and with at least one record of weight (kg) and length (cm) or height (m) were included in this study.

Children were excluded if they did not have information on the type and duration of breastfeeding recorded in the appropriate place in the Child Health programme or in free text in the clinical diary; if they had any type of coded pathology or if they had a record of social or environmental conditions that could limit the magnitude or duration of breastfeeding.

The inclusion criteria for the exclusive BF cohort were no record of infant formula for 6 months or using formula milk for less than 1 month. Inclusion criteria for the control cohort (non-exclusive BF) were a record/prescription of formula milk for more than 1 month, a record of hypogalactia in the mother, or a medical contraindication to BF. Patients in both cohorts were searched for an annual record of BMI (in kg/m^2^) up to the age of 10.

Cohorts were defined by the search keywords in the electronic health record. Keywords used to define the exclusive BF cohort were “leite mater”, “aleitamento mater”, “aleitamento excl”, “amamentaçao excl”, “materno excl”, and “mama excl”. The keywords used to define the control cohort were “supplement”, “leite formula”, and “leite adaptado”. The most frequent comorbidities of the parents of the children under study were also researched.

### 2.3. Exposure and Outcome Definitions

Exposure was defined as starting from the time point when BF was initiated and maintained until at least 6 months of age. The outcome of interest was defined as the attainment of obesity (BMI > P 97) or overweight (BMI: P 85–P 97) determined by the WHO percentile curves.

Time at risk was calculated from the index date (first appointment immediately after 6 months of age) until the development of the outcome of interest—overweight or obesity. The end date of the study period was censored if the child died, reached the outcome of interest, or ended follow-up. As the child’s diet evolved, they became eligible for the non-exclusive BF cohort, leaving the initial cohort to which they belonged.

Asthma and allergic rhinitis were used as positive controls for the results, since it has been described in literature that BF may be associated with a lower incidence of these diseases [24,25,26,27].

### 2.4. Statistical Analysis

Continuous variables were reported as the median and interquartile range (IQR), and categorical variables were presented as the absolute and relative frequencies.

To estimate the risk of developing overweight or obesity during the study period, cohorts were modelled using a Cox proportional hazards model. Hazard ratios (HRs) were established with 95% confidence intervals.

To correct the dependent observations resulting from having patients in more than one cohort at different points in time, the standard errors of the estimated hazard ratios (HRs) were adjusted to account for the correlations between those by applying the cluster method available in the R Survival package [28].

The propensity score (PS) was estimated using multivariable logistic regression with 15 covariates chosen a priori. Between-group differences in baseline characteristics were compared using standardised differences in both the unmatched and matched samples (differences > 10% were considered meaningful).

Children in the exclusive BF cohort were matched 1:1 with the control cohort (non-exclusive BF) based on gender and parental risk factors such as current, obesity, hypertension, hypercholesterolaemia, asthma, type 2 diabetes, gestational diabetes, social deprivation, psychosocial stress, psychiatric disorder major, sleep disorders, unspecified illness, chronic pain, and alcohol abuse. Propensity scores were calculated using a generalised linear model. The matching process was carried out using matching function software (MatchIt 4.5.5; R software 4.1.3). Standardised differences in patient characteristics post-matching were used to assess the adequacy of propensity score matching.

To obtain the annual incidence of obesity/overweight and HR values, only the matched group was included.

## 3. Results

### 3.1. Exposed Patient Characterisation

In total, 14,706 different children were eligible for this study: 6424 in the exclusive BF cohort and 8282 in the non-exclusive BF cohort. The median age was 2 years (IQR = 0) in both cohorts, with a predominance of patients aged between 1 and 2 years in the exclusive BF (n = 5148; 80.14%) and non-exclusive BF (n = 6592; 79.59%) cohorts.

There was a predominance of males in the exclusive BF (55.88%) cohort. The median weight at 2 years of age was greater in the exclusive BF cohort (P50 = 7; IQR = 6.70). A family history of a major psychiatric disorder was the most prevalent comorbidity in both cohorts (exclusive BF 20.52%; non-exclusive BF 31.49%), followed by a family history of hypercholesterolemia (exclusive BF 21.47%; non-exclusive BF 22.53%). A family history of alcohol abuse was the least prevalent comorbidity in both cohorts (exclusive BF 0.54%; non-exclusive BF 1.34%), as described in Table 1. The pairing was carried out in order to optimise the pairing of patients from the two cohorts in relation to as many variables as possible; however, it was not possible to find a pairing that guaranteed a SMD (Standardised Mean Difference) of less than 0.1 for all variables, while maintaining an acceptable number of patients in the post-pairing cohorts. However, Table 1 shows statistically significant higher rates of obesity and type 2 diabetes in the parents of the non-exclusive breastfeeding cohort.

After matching, 2614 patients were eligible for this study: 1309 for the exclusive BF cohort and 1305 for the non-exclusive BF cohort. Males predominated in both cohorts.

**Figure 1:**
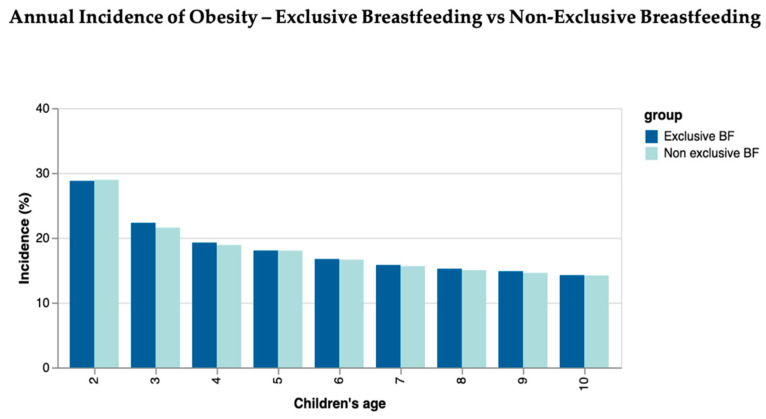
Annual incidence of obesity in exclusive BF children compared to non-exclusive BF children.

The annual incidence of obesity in both cohorts decreased as age increased. It was recorded as the highest at the age of 2, with 28.78% in the exclusive BF cohort and 28.94% in the non-exclusive BF cohort, although there were no clear statistical differences between the two groups at 2 years of age. The lowest rates were recorded at 10 years of age (Figure 1).

**Figure 2:**
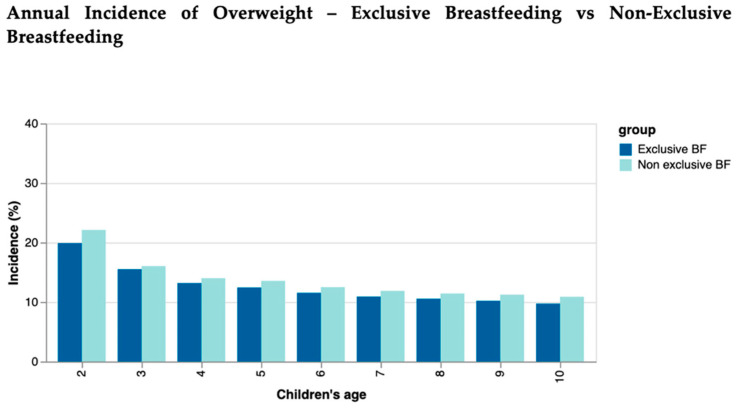
Annual incidence of overweight in exclusive BF children compared to non-exclusive BF children.

Throughout the study period, the annual incidence of overweight was higher in the non-exclusive BF cohort than in the exclusive BF cohort. The highest rates were also recorded at 2 years of age, with 19.89% in the exclusive BF cohort and 22.11% in the non-exclusive BF cohort. The lowest rates were recorded at 10 years of age, with a downward trend throughout the study period (Figure 2).

### 3.2. Family Risk Factors for the Development of Obesity and Overweight

**Figure 3:**
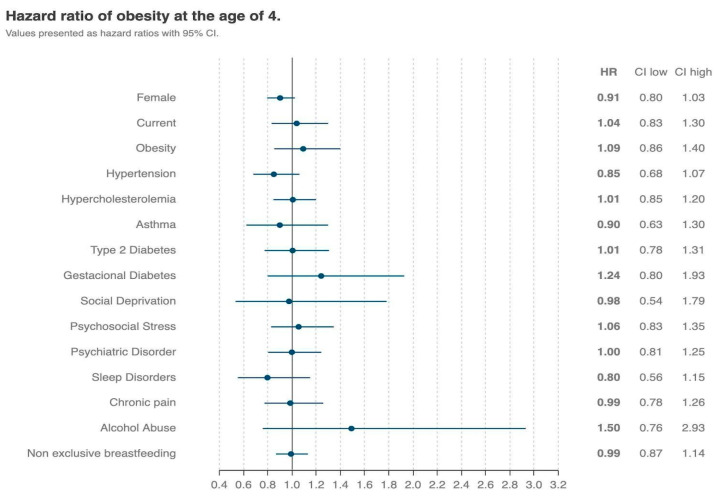
The family risk factors related to the development of obesity in exclusive BF children up to 6 months. Blue filled points indicate the risk ratios, blue lines indicate the 95% confidence intervals. CI—confidence interval; HR—hazard ratio.

Female children had a lower risk of developing obesity at the age of 4 when compared to their male counterparts (HR = 0.91, 95% CI = [0.80, 1.03]), but the confidence intervals showed no statistically significant differences. No association was found between family comorbidities and the development of obesity (Figure 3).

**Figure 4:**
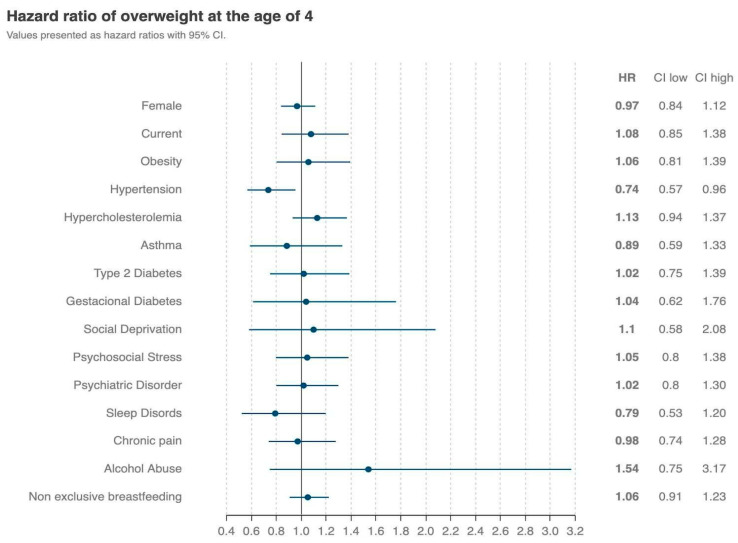
The family risk factors related to the development of overweight in exclusive BF children up to 6 months. Blue filled points indicate the risk ratios, blue lines indicate the 95% confidence intervals. CI—confidence interval; HR—hazard ratio.

Female children were also less at risk of developing overweight at 4 years of age (HR = 0.97, 95% CI = [0.84, 1.12]), but the confidence intervals showed no statistically significant differences. The only family comorbidity that seemingly presented an association with the development of overweight was hypertension (HR = 0.74, 95% CI = [0.57, 0.96]) (Figure 4).

### 3.3. Effect of BF on the Risk of Developing Obesity and Overweight over Time

**Figure 5:**
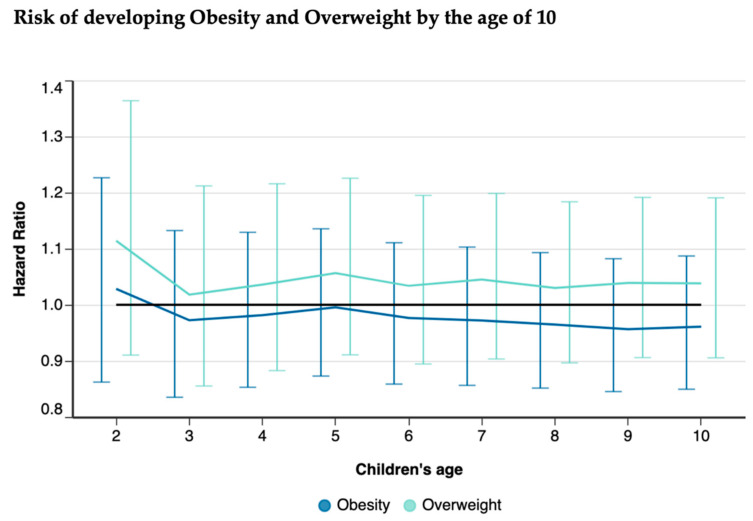
Comparison of risk of obesity and overweight among exclusive BF vs. non-exclusive BF children up to 10 years of age.

No significant differences between the effects of exclusive BF and non-exclusive BF were observed in the risk of childhood obesity or overweight for all follow-up periods (Figure 5).

**Figure 6:**
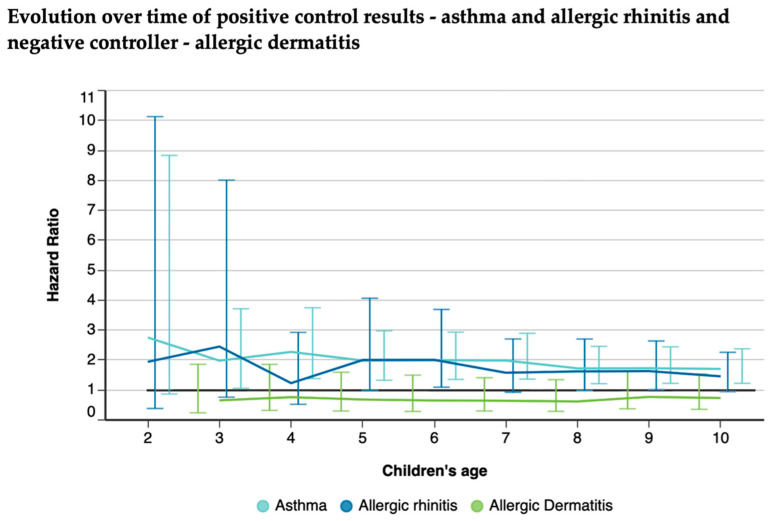
Risks of asthma, allergic rhinitis, and allergic dermatitis among exclusive BF vs. non-exclusive BF children up to 10 years of age.

Asthma and allergic rhinitis were used as positive control outcomes and allergic dermatitis as a negative control outcome (Figure 6). The other pathologies assessed and their results for each year can be found in Appendix A.

## 4. Discussion

The first result of our study was the similar trend in both cohorts of a reducing incidence of obesity and overweight in children over the period evaluated. While there were no major differences between the two cohorts in the incidence of obesity, when it came to reducing the incidence of overweight, the non-exclusive BF cohort had higher incidences at all the points assessed compared to the exclusive BF cohort, but this was not significant in either case.

These results are in line with the current literature. According to the COSI Portugal study, which assessed children aged 6 to 8 enrolled in the first and second years of school during the 2021/2022 school year, 31.9% of children were overweight, of which 13.5% were obese. Between 2008 (Round 1) and 2019 (Round 5), Portugal consistently showed an inverted trend in the prevalence of childhood overweight and obesity, but in 2022 (Round 6), this trend seemed not to be confirmed, with an increase of 1.6 percentage points (11.9% to 13.5%) in the prevalence of childhood obesity and 2.2 percentage points (29.7% to 31.9%) in the prevalence of childhood overweight. According to these results, Portugal is on par with the European average (29%), with one in three children being overweight. In 2022, the northern region had a prevalence of child overweight higher than that presented at the national level in COSI Portugal, with 31.9% [13].

However, despite the encouraging reduction, southern European countries maintain a high prevalence of overweight and obese children. This is attributed to the gradual shift from the healthy Mediterranean diet to a more Westernised diet and a decrease in physical activity. Current measures to fight childhood obesity and overweight should therefore be strengthened, requiring urgent and appropriate public health measures [13,29].

The predisposition to obesity and overweight in childhood, related to family risk factors, was also assessed in this study. From the results obtained, it seems that at the age of 4, female children have a lower risk of developing overweight and obesity. None of the family risk factors proved to be statistically significant for the development of obesity; however, for overweight, the family risk factor most likely to be related to its development was hypertension (HR = 0.74, 95% CI = [0.57, 0.96]). Studies have associated a familial predisposition to hypertension and other comorbidities with BMI in children, but more comprehensive studies are needed to specify the exact implications of a familial predisposition [30].

One of the goals of this study was to assess whether exclusive BF up to the age of 6 months reduced the risk of obesity or overweight up to the age of 10. This premise is consistent with the results of several studies that have shown a positive relationship between BF and anthropometric improvement in children, although there are some studies that are consistent with the results obtained in our study. Although it is possible to state with some degree of confidence that there appears to be no relationship between BF and obesity and/or overweight in children, it cannot be ruled out that this study lacked sufficient statistical power. This conclusion is not likely to be the result of a confounding effect because the methodology is robust and because the definition of the cohorts, despite being based on free text captured in clinical records, managed to capture signals that are known and with great strength.

Marseglia L. et al.’s meta-analysis, which highlights the current difficulty in identifying the mechanisms responsible for obesity, states that the duration of BF and maternal overweight before pregnancy seem to influence the inverse relationship between BF and obesity. However, the overall conclusion that can be taken from studies including this one is subject to controversy since these studies were often carried out on population groups with different inclusion and exclusion criteria, which makes it difficult to compare their results [31].

The study developed by Ramiro-Cortijo R. et al. tries to provide more information by analysing certain hormones in breast milk that appear to influence appetite. It compares mothers who went into full-term and premature labour, focusing on the association of that factor with growth during the first month of BF, and it evaluates the influence of the maternal nutritional status on the hormone levels in breast milk. When focusing only on the results for women with full-term labour, it was found that growth and weight were positively associated with insulin levels in breast milk, both in the first month of life and up to one year of age. Peptide YY levels were negatively associated with weight and the head circumference during the first month of life, due to the anorectic signal that peptide YY exerts by reducing appetite and, therefore, energy intake. The nutritional status and body composition of women in full-term labour were not associated with the hormone levels in breast milk. Regarding dietary patterns, in women in full-term labour, we did not detect any association with insulin levels. However, the fibre intake was negatively associated with peptide YY [32].

The PROBIT study also investigated the potential for the prevention of obesity and overweight in children up to the age of two by improving nutrition during childhood. The intervention group received information on protective behaviours, such as promoting BF. However, in the results of the study, it was not possible to detect a significantly lower prevalence of obesity in the intervention group [33].

Another randomised clinical trial assessed the effect of a pro-BF and healthy complementary feeding intervention aimed at teenage mothers and maternal grandmothers on growth and the prevalence of overweight and obesity in pre-school children. Although the intervention extended the duration of exclusive BF and delayed the start of complementary feeding, it had no impact on growth or the prevalence of overweight at ages 4 to 7 [34]. As such, the results showed no relationship between BF and childhood obesity and/or overweight. Those results can be considered robust since the possible limitations of the study were mitigated, and the signs described in the literature were found in the cohorts, namely asthma and allergic rhinitis placed as positive control results and allergic dermatitis as a negative control result.

A beneficial relationship between BF and a low risk of developing asthma in the future has been described in several studies. In a meta-analysis, it was shown that children fed with exclusive BF had a 19% lower risk of asthma compared to those who had less exclusive BF. Children who were exclusively breastfed for ≥6 months compared those for <6 months had a 30% lower risk of asthma. It was concluded that the duration and exclusivity of BF are associated with a lower risk of asthma in children aged <7 years [25].

The literature shows that never having been breastfed, compared to ever having been breastfed, is associated with a higher risk of asthma, as well as a shorter duration of BF [35]. In another cohort study, BF for at least 16 weeks, compared to no BF, was significantly associated with a lower prevalence of asthma from 3 to 8 years of age, with no evidence of attenuation of the association and regardless of a family history of allergies [36]. A randomised controlled trial concluded that withholding cow’s milk protein in infants at a high risk of allergy does not reduce the incidence of asthma. But it was found that children who were already breastfed had a lower incidence of wheezing than those who were not breastfed (59% and 74%, respectively) and that the effect persisted until 7 years of age [37]. The literature thus seems to agree with the results of our study, showing that BF is associated with a lower risk of asthma in the future.

The second positive control used in our study was allergic rhinitis, though this is not often described in the literature with statistically significant results. The exception was D. Codispoti et al., who sought to identify the environmental exposures and host factors that could cause allergic rhinitis during childhood at the age of 3. They concluded that among the various host factors, prolonged BF in African American individuals would reduce the risk of allergic rhinitis at 3 years of age [38]. 

In our study, atopic dermatitis was defined as a negative control. The effect of BF on atopic dermatitis remains subject to controversy according to the literature. A case–control study found a reduced risk of atopic dermatitis at 3 to 5 years of age in children who had never been breastfed and an increased risk of atopic dermatitis with an increasing duration of BF. However, while there are many benefits to BF, on the basis of this study, it is not possible to defend its recommendation for the prevention of atopic dermatitis [39].

## 5. Limitations

The population covered by the ULSM is essentially urban, with a wide range of health services on offer, which does not reflect the other regions of Portugal. The analysis was based on retrospective data from SClinico® clinical records, with a potential bias or residual confounding factors making causal inference difficult. In addition, this study only considered children up to the age of 10, leaving it impossible to understand the impact of BF in adolescence and young adults. This may limit a possible generalisation of the results to a wider population of patients with obesity or overweight. Furthermore, many clinical records lacked information on the type and duration of BF the child received. As the recording of these parameters depends on the medical record, this information was present in many clinical files but was not located in the appropriate place in the Child Health programme; instead, it was recorded in free text in the clinical diary, or in other cases, there was no such information. Moreover, this study was based on retrospective data, and data quality problems invariably arose. Additionally, modulated variables such as neonatal comorbidities and gestational age were not studied in this cohort and the small sample size limited the conclusions drawn in the study.

This study only compared children under exclusive and non-exclusive breastfeeding (mixed breastfeeding or milk formula), and there was no comparison with children who were only fed milk formula. This makes interpreting the data more difficult because detailed records of the percentage of calorific intake provided by breast milk were not available for the exclusively breastfed, non-exclusively breastfed and formula-fed groups, as well as the lack of information on other milk constituents.

So, to reduce bias, we performed PS-matching analysis on the variables gender, age, weight, and family risk factors, making the comparison between cohorts fairer; however, the loss of individuals after this process prevented the characterisation of participants other than those shown in Table 1 and likewise prevented them from being matched.

## 6. Conclusions

We found no relationship between exclusive BF vs. non-exclusive BF and the development of overweight or obesity in children up to the age of 10.

However, the incidence of obesity at the age of 2 was worryingly high, at 28.78% in the exclusive BF cohort and 28.94% in the non-exclusive BF cohort, along with that of overweight, at 19.89% in the exclusive BF cohort and 22.11% in the non-exclusive BF cohort.

This paradigm needs to be changed, and health professionals are a crucial ally in promoting and implementing healthy lifestyle habits among children’s parents.

## Figures and Tables

**Table 1:** Baseline characteristics of the unmatched and matched participant samples.

	Unmatched (n = 14,706)				Matched (n = 2614)				
	Exclusive Breastfeeding (n = 6424)		Non Exclusive Breastfeeding (n = 8282)		Exclusive Breastfeeding (n = 1309)		Non Exclusive Breastfeeding (n = 1305)		Mean Absolute Deviation
**Sex**									
Male—n (%)	3590	55.88%	3924	47.38%	838	64.02%	689	52.80%	0.19
Female—n (%)	2834	44.12%	4358	52.62%	471	35.98%	616	47.20%	0.19
**Parent Risk Factors—n (%)**									
Current	839	13.06%	1755	21.19%	51	3.90%	198	15.17%	0.29
Obesity	589	9.17%	988	11.93%	41	3.13%	155	11.88%	0.25
Hypertension	699	10.88%	1163	14.04%	143	10.92%	130	9.96%	0.03
Hypercholesterolemia	1379	21.47%	1866	22.53%	219	16.73%	261	20.00%	0.07
Asthma	241	3.75%	392	4.73%	21	1.60%	70	5.36%	0.16
Type 2 Diabetes	632	9.84%	998	12.05%	65	4.97%	110	8.43%	0.11
Gestational Diabetes	214	3.33%	218	2.63%	27	2.06%	32	2.45%	0.02
Social Deprivation	38	0.59%	274	3.31%	0	0.00%	21	1.61%	0.13
Psychosocial Stress	816	12.70%	1614	19.49%	182	13.90%	198	15.17%	0.03
Psychiatric Disorder Major	1318	20.52%	2608	31.49%	187	14.29%	324	24.83%	0.21
Sleep disorders	249	3.88%	356	4.30%	26	1.99%	69	5.29%	0.13
Unspecified illness	41	0.64%	100	1.21%	7	0.53%	25	1.92%	0.09
Chronic pain	319	4.97%	801	9.67%	110	8.49%	82	0.63%	0.37
Alcohol abuse	35	0.54%	111	1.34%	0	0.00%	15	1.15%	0.11

**Figure A1:**
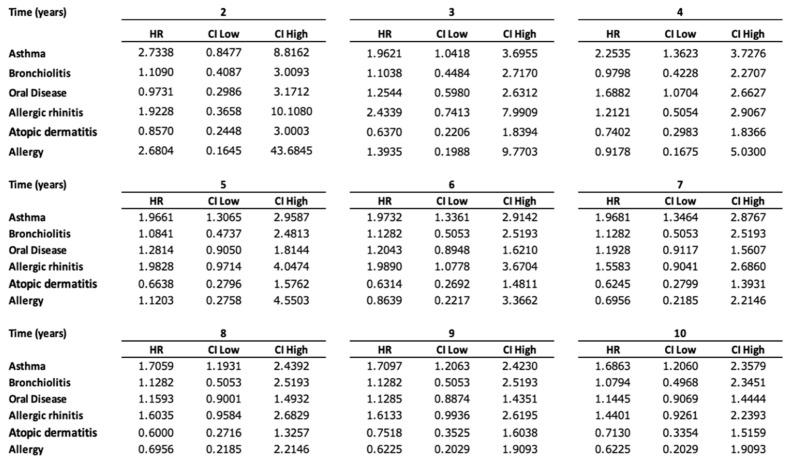
Other pathologies assessed in the study up to the age of 10, with their respective Hazard Ratio (HR).

## Data Availability

Data requests have to be submitted for ethical approval and authorisation by the hospital board.
